# Mechanisms of Bioactivities of Fucoidan from the Brown Seaweed *Fucus vesiculosus* L. of the Barents Sea

**DOI:** 10.3390/md18050275

**Published:** 2020-05-22

**Authors:** Olga N. Pozharitskaya, Ekaterina D. Obluchinskaya, Alexander N. Shikov

**Affiliations:** 1 Murmansk Marine Biological Institute of the Russian Academy of Sciences (MMBI RAS), Vladimirskaya, 17, 183010 Murmansk, Russia; olgapozhar@mail.ru (O.N.P.); okaterine@yandex.ru (E.D.O.); 2 St. Petersburg State Chemical Pharmaceutical University, Prof. Popov, 14, 197376 Saint-Petersburg, Russia

**Keywords:** fucoidan, cyclooxygenase, dipeptidyl peptidase IV, anti-inflammatory, anti-hyperglycemic, anti-coagulant, COVID19

## Abstract

The aim of this study was to elucidate some mechanisms of radical scavenging and the anti-inflammatory, anti-hyperglycemic, and anti-coagulant bioactivities of high molecular weight fucoidan from *Fucus vesiculosus* in several in vitro models. Fucoidan has displayed potent 1, 1-diphenyl-2-picryl hydrazil radical scavenging and reduction power activities. It significantly inhibits the cyclooxygenase-2 (COX-2) enzyme (IC_50_ 4.3 μg mL^−1^) with a greater selectivity index (lg(IC_80_ COX-2/IC_80_COX-1), −1.55) than the synthetic non-steroidal anti-inflammatory drug indomethacin (lg(IC_80_ COX-2/IC_80_COX-1), −0.09). A concentration-dependent inhibition of hyaluronidase enzyme with an IC_50_ of 2.9 μg mL^−1^ was observed. Fucoidan attenuated the lipopolysaccharide-induced expression of mitogen-activated protein kinase p38. Our findings suggest that the inhibition of dipeptidyl peptidase-IV (DPP-IV) (IC_50_ 1.11 μg mL^−1^) is one of the possible mechanisms involved in the anti-hyperglycemic activity of fucoidan. At a concentration of 3.2 μg mL^−1^, fucoidan prolongs the activated partial thromboplastin time and thrombin time by 1.5-fold and 2.5-fold compared with a control, respectively. A significant increase of prothrombin time was observed after the concentration of fucoidan was increased above 80 μg mL^−1^. This evidenced that fucoidan may have an effect on intrinsic/common pathways and little effect on the extrinsic mechanism. This study sheds light on the multiple pathways of the bioactivities of fucoidan. As far as we know, the inhibition of hyaluronidase and DPP-IV by high molecular fucoidan was studied for the first time in this work. Our results and literature data suggest that molecular weight, sulfate content, fucose content, and polyphenols may contribute to these activities. It seems that high molecular weight fucoidan has promising therapeutic applications in different pharmacological settings. Anti-oxidant, anti-inflammatory and anti-coagulant drugs have been used for the management of complications of COVID19. Taken as a whole, fucoidan could be considered as a prospective candidate for the treatment of patients with COVID19; however, additional research in this field is required.

## 1. Introduction

Natural sources such as marine flora are of interest in the development of new effective medicines. In this context, marine algae represent one of the richest sources of bioactive compounds [1]. Fucoidans are biologically active sulfated polysaccharides that are synthesized by brown algae. Their analogues have not yet been found in terrestrial organisms. In recent years, fucoidans have become the subject of increased attention and numerous studies. They represent an extensive class of biopolymers, the content and structure of which varies depending on the type of algae, their places of growth, harvest season and other factors [2,3,4,5]. Fucoidan is renowned as a substance with a broad spectrum of biological activities including anti-oxidant [6,7,8], anti-inflammatory [9,10], anti-diabetic [11,12], anti-coagulant [13,14,15], anti-thrombotic [16], and anti-viral [17] activity, etc., and has been used for oral [16,18,19,20] and topical [21,22] applications.

It is reported that brown seaweed is resistant to oxidative stress under salt conditions; the absence of oxidative damage in biochemical parameters caused by stress suggests that they contain anti-oxidant metabolites [23]. Chronic oxidative stress has been shown to play an important role in the occurrence of chronic diseases such as inflammatory pathogenesis and type 2 diabetes [24].

One of the possible mechanisms of the anti-inflammatory action of various drugs is the inhibition of the arachidonic acid cascade and the synthesis of prostaglandins and leukotrienes [25]. Cyclooxygenase 1/2 (COX-1/COX-2), key enzymes of this cascade, are promising targets for anti-inflammatory drugs [26]. The synthesis of pro-inflammatory cytokines is initiated by intracellular signaling, one of the links of which involves mitogen-activated protein kinases (MAPK). When cells are stimulated with inflammatory factors, MAPK are phosphorylated and activate factors that trigger the transcription of inflammatory mediators (including interleukins, COX-2, arachidonic acid, etc.) [27,28]. Hyaluronidase is an enzyme located in connective tissues which plays an important role in the development of inflammatory diseases, since the destruction of the connective tissue promotes the spreading of chemotactic factors of inflammation [29]. The inhibition of hyaluronidase prevents tissue damage and is capable of suppressing brain metastasis [30]. Therefore, it is reasonable to study the effects of fucoidan on the COX-1/2 enzymes, MAPK, and hyaluronidase.

Insufficient glycemic control of patients with diabetes is one of the leading causes of death. Dipeptidyl peptidase-IV (DPP-IV) is an enzyme that is involved in the inhibition of the rapid degradation of incretin hormones, which prevents postprandial hyperglycemia. Inhibiting DPP-IV prolongs the action of incretins, which reduces glucose production and increases insulin production [31]. Anti-hyperglycemic effects of fucoidans have been reported in the literature [32]. DPP-IV inhibitors have been investigated as a new therapy; however, the mechanisms of fucoidan have been not proven.

Due to its anti-coagulant properties, fucoidan has gained increasing attention from scientists. The mechanisms of anti-coagulant action of fucoidan are predominantly related to the in vitro potentiation of the activated partial thromboplastin time (APTT), thrombin time (TT), and prothrombin time (PT). Fucoidans with low and middle molecular weight have been the focus of several studies [33,34,35], while little is known about high molecular weight fucoidans.

A number of studies and reviews have summarized the biological activities of fucoidans; however, these polysaccharides are heterogeneous, and their structure and activity vary depending on the source, processing techniques, molecular weight, etc. [7,19,36,37]. The molecular weight of fucoidans shows a vast divergence from 43 up to 1600 kDa [38]. The variation between fucoidans is reflected in sulfate content (9%–40%), fucose (25%–93%), uronic acid (up to 25%), and neutral sugars [3]. Thus, each fucoidan isolated from a seaweed is a compound with a unique structure and consequently has potential specific biological activities. The majority of articles have focused on low molecular weight fucoidans.

As a continuation of our exploration of fucoidan recovered from *Fucus vesiculosus* L. of the Barents Sea, the aim of this study was to elucidate some mechanisms of radical scavenging and the anti-inflammatory, anti-hyperglycemic, and anti-coagulant bioactivities of high molecular weight fucoidan from *Fucus vesiculosus* of the Barents Sea in several in vitro models. As far as we know, the inhibition of hyaluronidase and DPP-IV by high molecular fucoidan was studied for the first time in this work.

## 2. Results and Discussion

The radical scavenging and the anti-inflammatory, anti-hyperglycemic, and anti-coagulant activities of fucoidan from *F. vesiculosus* were evaluated using different in vitro assays.

### 2.1. Radical Scavenging Activities

**Figure 1:**
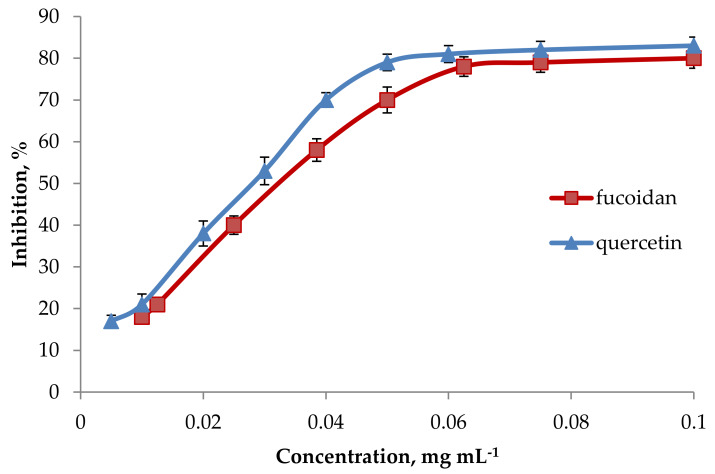
DPPH radical scavenging activity for fucoidan. Each value represents the mean ± SD of three determinations.

The 1, 1-Diphenyl-2-picryl hydrazil (DPPH) radical-scavenging model is one of the convenient tools for estimating the free radical-scavenging activities. The radical scavenging capacity of fucoidan arises from its ability to donate hydrogen atoms towards the DPPH free radical (purple), thereby forming DPPH-H (yellow) [14]. We have found that the scavenging ability of fucoidan was concentration-related (Figure 1). The IC_50_ of fucoidan was equal to that of quercetin (Table 1). A similar result was observed for the ascorbic acid equivalent anti-oxidant capacity (AEAC) for fucoidan and quercetin. Flavonoids are also electron donors, and electron donation is mainly derived from the flavonoid’s B-ring [39]. Therefore, quercetin was used as a standard anti-oxidant in this test. Thus, fucoidan has a lower free-radical scavenging activity than that of synthetic anti-oxidants, but its activity is comparable to that of the natural anti-oxidant quercetin, especially at concentrations above 0.06 mg mL^−1^ (Figure 1).

A similar specificity was described for fucoidans from *Undaria pinnatifida*. Crude fucoidan (sulfate content 23%, fucose 29 mol%, xylose 30 mol%, galactose 23 mol%, glucose 3 mol%, uronic acid 4 mol%) and fucoidan with a molecular weight (Mw) cut off of 300 kDa (sulfate content 20%, fucose 27 mol%, xylose 3 mol%, galactose 18 mol%, glucose 2 mol%, uronic acid 4.6 mol%) exhibited significantly lower antioxidant activities (7.4 μg mL^−1^ and 9.0 μg mL^−1^ trolox equivalent, respectively) compared to both ascorbic acid (AA) and butylated hydroxyanisole (BHA) (244 μg mL^−1^ and 235 μg mL^−1^ trolox equivalent, respectively) in DPPH assay [8]. The same trend was observed for fucoidan from brown seaweed *Sargassum binderi* with an Mw cut off of 2000 Da [40]. This fucoidan was less potent in the scavenging of DPPH (IC_50_ 2.0 mg mL^−1^) when compared with our fucoidan, while the IC_50_ value reported by authors for BHA, butylated hydroxytoluene (BHT), and AA are in agreement with our data (Table 1).

It is noteworthy that the activity of our fucoidan was much higher in DPPH assay than the activity of fucoidan (Mw 34.4 kDa, sulfate content 27.1%, fucose 41.2 mol%, galactose 6 mol%, glucose 6 mol%, xylose 15 mol%, mannose 11.3 mol%, uronic acid 24.6 mol%) from *Ascophyllum nodosum* (30.4% scavenging of DPPH at 10 mg mL^−1^) [41] or fucoidan with unknown molecular weight (sulfate content 21.2%, fucose 76.8 mol%, galactose 23.2 mol%, total phenolics 5.6%) from *F. vesiculosus* (23% scavenging of DPPH at 1 mg mL^−1^) [42].

**Figure 2:**
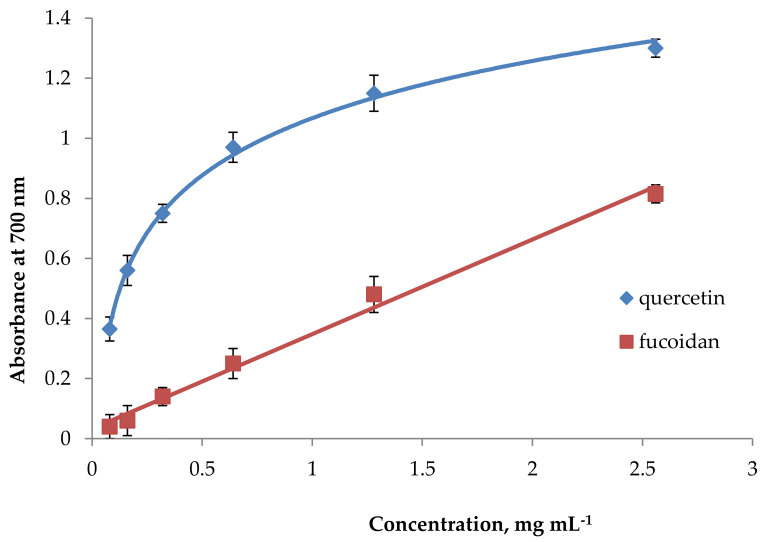
Reducing power for fucoidan. Each value represents the mean ± SD of three determinations.

In the reducing power assay, the absorbance increased linearly with the concentration of fucoidan (Figure 2). This evidences its ability to donate electrons. Since the electron acceptor (Fe^3+^(CN^−^)_6_) was added to excess in the assay method, the range of concentrations (0–2.5 mg mL^−1^ reaction) was chosen so that the absorbance did not exceed 1.5 U, as a higher absorbance decreases the accuracy due to the logarithmic relation between absorbance and transmittance [43]. From the absorbance plot, an arbitrary point at RC_0.5AU_ (reducing capacity at 0.5 absorbance unit) could be obtained to indicate the potency of a test substance. This is a convenient point for comparison with an electron-donating anti-oxidant, such as quercetin (Figure 2), to obtain a quercetin equivalent reducing capacity (QERC) value. The RC_0.5AU_ values were 1.48 mg mL^−1^ for fucoidan and 0.11 mg mL^−1^ for quercetin. The QERC value for fucoidan was 7432 ± 62. The reducing power of fucoidan from *Sargassum binderi* (0.6 mg gallic acid equivalents (GAE) 100 g^−1^) was significantly lower than that of BHA (63.6 mg GAE 100 g^−1^), BHT (32.4 mg GAE 100 g^−1^), or AA (42.3 mg GAE 100 g^−1^) [40].

The reducing power of fucoidans from *Durvillaea antarctica* (Mw 482 kDa, molar rate 0.2, 26.2, 0.9, 2.9, and 2.7 for fucose, glucose, xylose, galactose, mannose, and sorbose, respectively) and from *Ulva lactuca* (Mw 466 kDa, molar rate 1.1, 1.9, 0.2, 0.5, 0.3, 6.7, and 0.5 for fucose, glucose, galactose, arabinose, xylose, mannose, and sorbose, respectively) were approximately two folds lower than for our fucoidan [44]. It should be noted that the reducing power of fucoidan from *Ascophyllum nodosum* at the concentration of 3 mg mL ^−1^ [41] was equal to the reducing power of our fucoidan at the same concentration. A noteworthy radical scavenging potential (IC_50_ 0.045 mg mL^−1^ in DPPH assay and 399.35 μg mg^−1^ ascorbic acid equivalent in reducing power) was found by Phull et al. for commercial fucoidan from *Undaria pinnatifida* [45]. However, the authors have not provided information about the Mw and composition for this fucoidan.

The factors determining the radical scavenging activity of fucoidan are comprehensive, and there is not a single factor [7,46]. He et al. observed that sulfated polysaccharides from different seaweeds with an Mw of 404–482 kDa and total sugar content 23%–64% were more active radical scavengers than the non-sulfated polysaccharide with an Mw of 591 kDa [44]. The anti-oxidant activities of sulfated polysaccharides from *Corallina officinalis* show a positive correlation with the sulfate content [47]; the sulfate content may be affected by purification techniques [48]. Yuan and Macquarrie demonstrated that the reducing power of fucoidan was increased with higher sulfate content and molecular weight [41]. However, the relationship between molecular weight and radical scavenging activities is not simply linear [49].

The sulfate content and molecular weight are not the only factors influencing radical scavenging activity. It was reported that crude fucoidans in the presence of polyphenols had a stronger radical scavenging activity than purified fucoidan [49,50,51]. A high and significant correlation between phenolics content and DPPH radical scavenging activity was demonstrated for Icelandic seaweed extracts [52]. Potent radical scavenging derivatives of phloroglucinol were isolated from ethanol extract of *F. vesiculosus*. Trifucodiphlorethol A, trifucotriphlorethol A, and fucotriphlorethol A showed inhibition of DPPH with an IC_50_ of 10.0–14.4 µg mL^−1^ [53].

Our results suggest that high molecular weight fucoidan from *F. vesiculosus* is a good radical scavenging agent. This activity could be related to its molecular weight and sulfate and polyphenol contents.

### 2.2. Anti-Inflammatory Activities

The anti-inflammatory activity and associated molecular mechanisms of fucoidan have gained significant attention [45]. In this study, the anti-inflammatory properties of fucoidan were assessed in different test systems such as by the inhibition of COX, hyaluronidase, and MAPK p38 enzymes.

**Figure 3:**
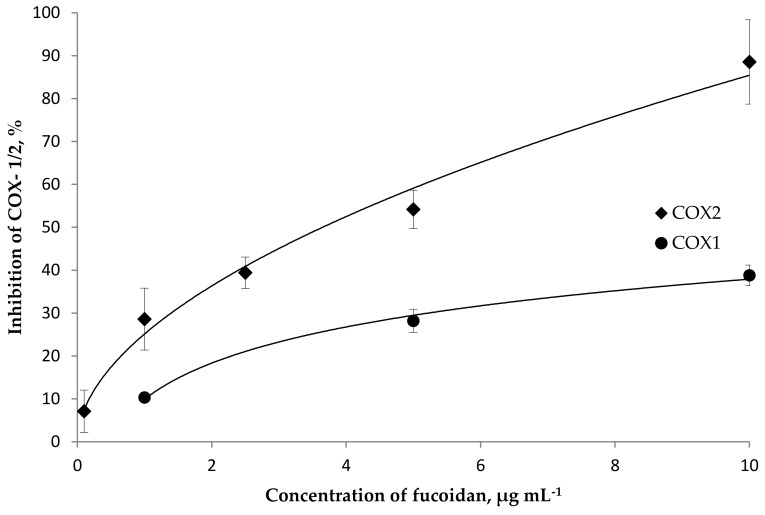
The inhibitory effect of fucoidan on the cyclooxygenase (COX)-1 and COX-2 enzymes. Each value represents the mean ± SD of three determinations.

Our efforts commenced by evaluating the inhibitory activity towards both isoforms of pro-inflammatory cyclooxygenase (COX-1 and 2) enzymes. It was found that fucoidan concentration-dependently inhibits the activity of both isoforms of the COX enzyme in a concentration range of 0.1–10 μg mL^−1^ (Figure 3). Fucoidan exhibited potent COX-2 inhibitory activity (IC_50_ 4.3 μg mL^−1^), which was found to be higher than the level of COX-1 inhibition (IC_50_ 27 μg mL^−1^). A higher selectivity index (lg IC_80_ COX-2/COX-1 −1.55) was calculated for fucoidan than for the reference drug indomethacin (lg IC_80_ COX-2/COX-1 −0.09). COX-1 is expressed constitutively in almost all cell types. COX-1 controls the production of prostaglandins, which regulate the integrity of the gastrointestinal mucosa, platelet function and renal blood flow, and COX-2 is involved in the synthesis of prostaglandins involved in inflammation. Moreover, COX-2 is not present under normal conditions, but is formed under the influence of some tissue factors initiating the inflammatory reaction [54,55]. It is assumed that the anti-inflammatory effect of non-steroidal anti-inflammatory drugs is associated with the inhibition of COX-2, and their adverse side effect is associated with the inhibition of COX-1. In this regard, fucoidan from *F. vesiculosus* is promising.

The inhibition of COX enzymes by fucoidan was reported previously in cell lines [56] and suggested in silico [57]. According to in silico calculations, the bonding energy required by fucoidan binding to COX-1 and COX-2 was almost half of that required by acetylsalicylic acid. This represents evidence for the high potential for fucoidan as an inhibitor of COX-1 and COX-2 [57]. In most studies, the inhibition of COX-2 by fucoidan was observed. As a result of the study of three fractions of fucoidan from *Ecklonia cava*, it was found that fraction F3 (with a low Mw of 88.3 g mol^−1^, high sulfate content of 39.1%, high fucose 77.9 mol%, rhamnose 2.3 mol%, galactose 10.1 mol%, glucose 2.2 mol%, xylose 7.5 mol%) was a very strong inhibitor of NO by down-regulating the expression of iNOS, COX-2, and pro-inflammatory cytokines in raw 264.7 murine macrophage cells stimulated with lipopolysaccharide (LPS). The authors believe that the level of NO released from macrophages was proportionally related to the Mw and sulfate content of the fucoidan [58]. Commercial fucoidan from *Undaria pinnatifida* (with high sulfate and fucose content, and unknown Mw) at the concentrations of 0–100 μg mL^−1^ down-regulated the expression of COX-2 in a concentration and time-dependent manner in rabbit articular chondrocytes [59]. It should be noted that this fucoidan showed a radical scavenging activity very similar to that exhibited by our fucoidan from *F. vesiculosus* (Section 2.1).

The alcohol-induced up-regulation of COX-2 in murine liver was markedly attenuated in the presence of commercial fucoidan from *F.* *vesiculosus* (purity > 95%, with peaks of Mw 680 kDa, 190 kDa, and 6.6 kDa, molar rate 1:0.03:0.02:0.04:0.2:1.2 for fucose, galactose, mannose, xylose, uronic acid, and sulfate, respectively). The effective doses were 30–60 mg kg^−1^ [60]. The fucoidan fraction from *Turbinaria ornata* (sulfate content 38.3%, polyphenols 0.25%, Mw not reported, fucose 86.4 mol%, rhamnose 0.4 mol%, galactose 1.7 mol%, glucose 0.8 mol%) concentration-dependently (25–100 µg mL^−1^) suppressed the expression of COX-2 and pro-inflammatory cytokines in LPS-induced RAW 264.7 macrophages [61].

Recently, the inhibition of COX-1/2 (human) by fucoidan from different sources was investigated in vitro using AChE competitive ELISA [20]. Commercial crude fucoidan from *F.vesiculosus* (Mw 20–200 kDa, sulfate content 23%, fucose 33 mol%, uronic acid 8 mol%) significantly inhibited COX-1 activity with IC_50_ 22.8 ng μL^−1^ but did not attenuate COX-2. The fucoidan extract with a low Mw from *Cladosiphon novae-caledoniae* (sulfate content 14.5%, fucose 73 mol%, xylose 12 mol%, mannose 7 mol%) inhibited COX-1 and COX-2 with IC_50_ values of 19.35 ng μL^−1^ and 80.64 ng μL^−1^, respectively. In the same experiments, commercial purified (>95%) fucoidan from *F. vesiculosus* with a peak Mw of 68.6 KDa did not show a COX-1/2 inhibitory effect. The authors of this study speculated that the purification of fucoidan could lead to a decrease of sulfating and resulted in a decrease of COX inhibition [20]. This hypothesis, as well as the results of previously described studies [58,59], is consistent with our above-mentioned results showing the promising inhibition of both COX-1 and COX-2 by fucoidan from *F. vesiculosus* with a sulfate content of 27%. It is noteworthy that all discussed fucoidans were rich in fucose. The polyphenols presented in our fucoidan could contribute to COX inhibition as well. In particular, phloroglucinols from an ethanol extract of *F. vesiculosus* inhibited COX-1 with an IC_50_ of 39–44 µg mL^−1^ [53].

The anti-hyaluronidase activity of fucoidan from *F. vesiculosus* was compared with the officinal medicine Alflutop^®^ (Biotehnos S.A, Otopeni, Romania) which is recommended as a chondroprotective drug with anti-hyaluronidase and anti-inflammatory activity [62]. Alflutop is a bioactive complex from small fish species which contains glycosaminoglycans, essential amino acids, essential fatty acids, glycerophosphates, and microelements [63]. Both fucoidan and the positive control Alflutop have been shown to inhibit hyaluronidase enzyme activity in a concentration-dependent manner. The IC_50_ value for the fucoidan was 2.9 μg mL^−1^, while that for Alflutop was 3.3 μg mL^−1^. Fucoidan isolated from *Undaria pinnatifida* was capable of inhibiting hyaluronidase activity in a concentration-dependent manner with an IC_50_ of 13.0 μg mL^−1^. This fucoidan had a molar composition of fucose: galactose: glucuronic acid: sulfuric acid of 1.0:1.0:0.04:5.2 [29]. Our fucoidan from *F.* *vesiculosus* contained fucose, glucose, galactose, xylose, mannose, and arabinose at a molar ratio of 1.0:0.16:0.05:0.09:0.03:0.03, respectively [18]. We assumed that the significant contribution to the anti-hyaluronidase activity of fucoidan could be attributed to fucose.

In the next stage of our study, the anti-inflammatory effect of fucoidan extracted from *F. vesiculosus* was assessed in human mononuclear U937 cells. The stimulation of U937 with LPS resulted in the direct activation of MAPK p38. The results presented in Table 2 indicate that MAPK p38 was concentration-dependently inhibited by fucoidan. Interestingly, fucoidan was a more potent inhibitor of MAPK p38 than the specific inhibitor SB203580.

Our results are consistent with previous studies demonstrating that commercial fucoidan purified from *Fucus vesiculosus* (composition is not provided) significantly suppresses the LPS-induced release of NO, prostaglandin E₂ (PGE2), monocyte chemoattractant protein (MCP-1), interleukin (IL)-1β, and tumor necrosis factor (TNF)-α by the inhibition of nuclear factor kappa B (NF-κB), Akt, extracellular signal-regulated kinase (ERK), MAPK p38, and c-Jun N-terminal kinases (JNK) activation in BV2 microglia cells [56]. Additionally, the MAPK pathway is an insulin signaling cascade. Commercial fucoidan from *Fucus vesiculosus* (composition not provided) inhibits adipocyte differentiation though the MAPK p38 pathway in 3T3-L1 preadipocytes [64]. Fucoidan-like crude polysaccharide from *Sargassum horneri* (sulfate content 12%, polyphenols 3.9%) attenuates the phosphorylation of MAPK p38 at a concentration of 100 µg mL^−1^ [65].

Taken together, these results and literature data suggest that fucoidan has significant anti-inflammatory activity that involves different pathways: the inhibition of COX-1/2, hyaluronidase and MAPK p38. Apparently, the sulfate content, fucose content, and polyphenols may contribute to these activities.

### 2.3. Anti-Hyperglycemic Activity

We have found a concentration-dependent inhibition of the enzyme DPP-IV by fucoidan at the concentration range of 0.02–200 μg mL^−1^. The IC_50_ was 11.1 μg mL^−1^ and the maximum inhibition degree was 60%–75%, while for the reference preparation of sitagliptin, the IC_50_ was 3.8 μg mL^−1^. In recent years, the DPP-IV enzyme has become an important new drug target for diabetes, and the efforts of the pharmaceutical industry have led to the development of DPP-IV inhibitors with good safety and efficacy profiles. There are few data regarding the inhibitory effect of brown algae extracts on DPP-IV activity. The value of IC_50_ for methanol and acetone extracts from *Turbinaria ornata* seaweed was 55.2 μg mL^−1^ [66]. A similar activity was also demonstrated for methanol extracts of algae *Sargassum polycystum* and *S. wightii*: IC_50_ values were 38.3 and 36.9 μg mL^−1^, respectively [67]. However, the polysaccharide content in these extracts was not provided.

As far as we know, our findings suggest for the first time that the inhibition of DPP-IV is one of the possible mechanisms involved in the anti-hyperglycemic effect of fucoidan.

### 2.4. Anti-Coagulant Activity

**Figure 4:**
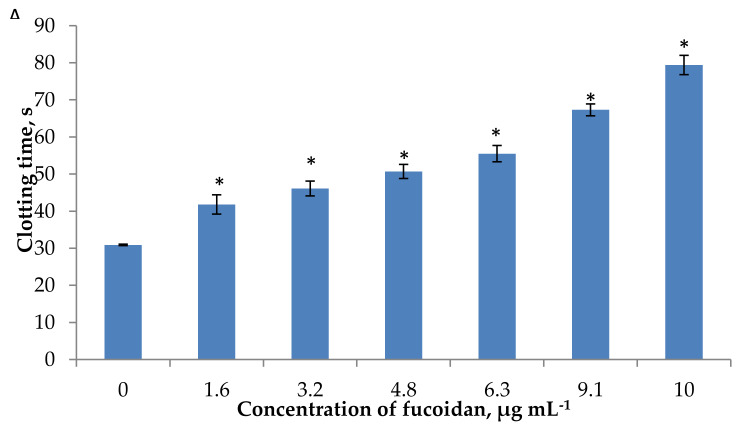

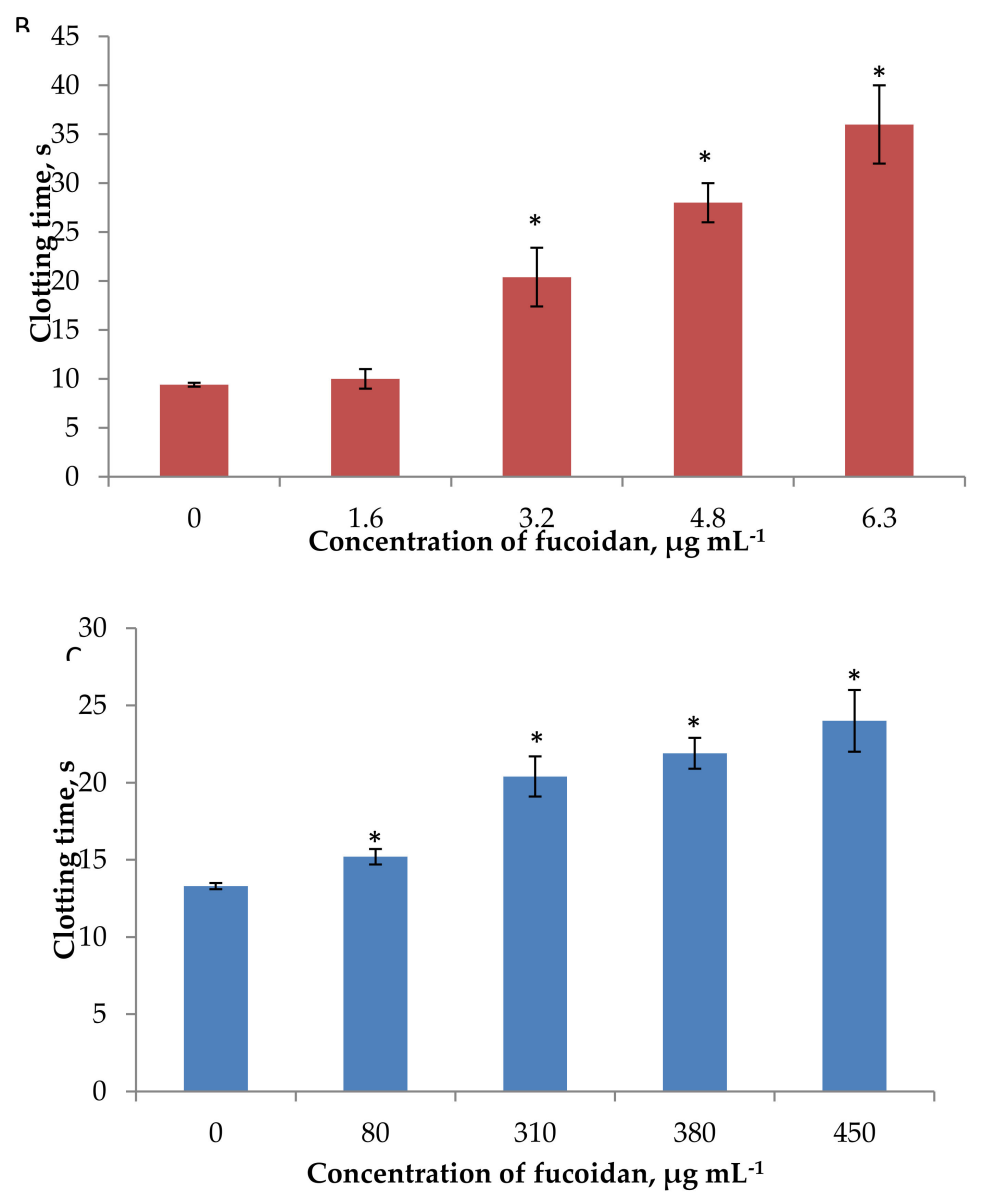
Anti-coagulant activity of fucoidan measured by activated partial thromboplastin time (APTT) (**A**), thrombin time (TT) (**B**), and prothrombin time (PT) (**C**) assays. Each value represents the mean ± SD of three determinations. * differences are statistically significant compared with the control, Student’s *t*-test *p* < 0.05.

The anti-coagulant activity of fucoidan was determined by activated APTT, TT, and PT assays in vitro that characterized two pathways of the coagulation process. The intrinsic pathway was mainly evaluated by the APTT assay, whereas in the PT assay, coagulation was triggered through the extrinsic pathway [68]. These pathways merge at the common point in which prothrombin is converted to thrombin, which leads to the formation of fibrin threads [34]. Fucoidan induced the concentration-dependent prolongation of APTT, TT and PT (Figure 4A–C). The effect of fucoidan on APTT and TT was more pronounced than on PT. In APTT assay, fucoidan at the concentration of 3.2 μg mL^−1^ extended the clotting time by up to 46.1 s (by 1.5-fold longer compared with the control) (Figure 4A). This was equal to the effect of 0.048 IU mL^−1^ heparin (clotting time 44.5 s). The maximal prolongation by 2.5-fold was observed for fucoidan at 10 µg mL^−1^. Apparently, our fucoidan was more active than fucoidan isolated from cloned *Grateloupia filicina* (Mw 11.7 kDa, sulfate content 19.9%, molar rate 0.11:0.08:1.0:0.01:0.11:0.02 for fucose, glucose, galactose, mannose, xylose, and glucuronic acid, respectively) which increased APTT by 2.5-fold compared to the control at the concentration of 30 µg mL^−1^ [69]. Similar patterns in clotting time were observed regarding the effect of our fucoidan on TT, where fucoidan (3.2 μg mL^−1^) extended the clotting time by 2.2-fold compared with the control (Figure 4B). Our results are consistent with those reported for fucoidan from *F. vesiculosus* (Mw 150 kDa, sulfate content 9.1%, fucose 92.5 mol%, galactose 7.5 mol%) [33], fucoidan from *F. vesiculosus* (Mw 98 kDa, sulfate content 9.8%, fucose 86.8 mol%, galactose 9.0 mol%, xylose 4.2 mol%) [34], and fucoidans from *Grateloupia filicina* (Mw 11.7 kDa [69] and Mw 1.4 kDa) [70], which suggested that anti-coagulant activities mainly resulted from the inhibition of the intrinsic coagulation pathway. An increase in APTT may also be the result of the inhibition of enzymes and cofactors of the common pathway, and not only the intrinsic pathway.

It is obvious from Figure 4C that fucoidan was not able to prolong PT significantly. The clotting time at 80 μg mL^−1^ of fucoidan was prolonged from 13.3 s (control) up to 15.2 s. The same clotting time (15.9 s) was observed for heparin at 0.33 IU mL^−1^. The increase of fucoidan concentration above 80 μg mL^−1^ led to a greater increase in PT. These observations are of a similar magnitude to those by Irhimeh et al. [13], who reported that fucoidan from *Undaria pinnatifida* (sulfate content 29.0 mol%, fucose 24.8 mol%, galactose 20.4 mol%) at low concentrations (up to 63 mg L^−1^) had no effect on PT in vitro; however, with an increase of concentration above 125 mg L^−1^ the PT was increased. A comparison of the effect of heparin and fucoidan allows us to calculate that the activity of 1 mg of fucoidan corresponds to the activity of 2.7 IU of heparin on PT and 14.1 IU on APTT, respectively.

The anti-coagulant activity of fucoidan was confirmed in clinic. The oral administration of fucoidan from *Undaria pinnatifida* by volunteers (3 g for 12 days) resulted in an increase of APTT from 28 s up to 34 s, whereas TT was decreased from 18.6 s to 17.5 s (*p* < 0.02), and PT was not changed statistically significantly [13].

Thus, fucoidan from *F. vesiculosus* showed potent anti-coagulant activities. It prolongs APTT and TT significantly and concentration-dependently and prolongs PT at high concentrations. This showed that the studied fucoidan may have an effect on intrinsic/common pathways and little effect on the extrinsic mechanism. The activity of our fucoidan with high molecular weight and sulfate content is in line with the literature data, which indicated that the higher anti-coagulant activity of fucoidans is correlated with high sulfate content and a large Mw [71].

## 3. Materials and Methods

### 3.1. Materials

#### 3.1.1. Fucoidan

Fucoidan with an average Mw of 735 kDa was provided by MMBI KSC RAS, Murmansk, Russia. Fucoidan was extracted from *F. vesiculosus* by ultrasound-assisted extraction [72] according to the procedure described previously [18]. The yield of fucoidan was 4.9% by mass. Fucoidan contained 79.5% of neutral carbohydrates, 27.0% of sulfate residues, and 0.7% of uronic acid. Carbohydrates were represented by fucose (73.5 mol%), glucose (11.8 mol%), galactose (3.7 mol%), xylose (6.6 mol%), mannose (0.2 mol%), and arabinose (0.2 mol%). The molar ratio of fucose, glucose, galactose, xylose, mannose, and arabinose was 1.0:0.16:0.05:0.09:0.03:0.03, respectively, as evidenced by high performance liquid chromatography (HPLC) [18]. According to the literature data, fucoidans from *Fucus* spp. are represented mainly by (1→3)- and (1→4)-linked α-l-fucopyranose residues [3,36]. Electrospray ionization mass spectrometry evidenced that sulfate groups are attached mostly at C-2 and sometimes at C-4 of sugar residues in fucoidans from brown seaweeds [73].

The concentration of total polyphenols was determined with Folin–Ciocalteu reagent as described previously [74]. Phloroglucinol (Sigma, St. Louis, MO, USA) was used as reference. The total polyphenol content was 4.7 ± 0.5 mg PGEq g^−1^ (milligram of phloroglucinol equivalent PGEq per 1 g fucoidan).

#### 3.1.2. Chemicals

Analytical-grade chemicals and solvents for extraction and chromatography were purchased from local chemical suppliers. Specialized chemicals and reference compounds were purchased from Sigma Chemical Co. (St Louis, MO, USA) and Cayman Chemical Co. (Ann Arbor, MI, USA). Reagents for activated partial thromboplastin time (APTT), prothrombin time (PT), thrombin time (TT), and reference normal human platelet-poor plasma (RNP) (cat. No717) were purchased from LLC Technology-Standard, Barnaul, Russia. RNP plasma was restored with water and kept at room temperature for 15–20 min before assay. Deionized water, filtered through a 0.22 µm filter, was used to reconstitute the calibrators and reagents.

### 3.2. Radical Scavenging Activity Assays

#### 3.2.1. 1, 1-Diphenyl-2-picryl hydrazil Radical Scavenging Activities

The DPPH radical scavenging activity was determined according to the well accepted procedure. In brief, fucoidan solution (50 µL in water) was mixed with 50 µL methanol and DPPH solution (100 µL), vortexed, and kept in the dark at room temperature for 30 min. The decrease in absorbance of the mixture was measured at 517 nm against a reagent blank. The DPPH tests were performed in triplicate. The percentage of inhibition was calculated as follows:

Inhibition (%) = (A−C)/A × 100(1)

where A is the absorbance of the control, and C is the absorbance of the sample.

The inhibitory concentration (IC_50_) or the concentration of the title compound at which it scavenges 50% of radical activities was calculated from the plot with concentrations of the sample against the percentage of inhibition and expressed in milligrams per milliliter. The IC_50_ of fucoidan was calculated and compared with that of butylated hydroxyanisole (BHA), butylated hydroxytoluene (BHT), quercetin and ascorbic acid (AA).

The DPPH radical scavenging was then expressed in terms of the ascorbic acid equivalent anti-oxidant capacity (AEAC), which was calculated based on the equivalent anti-oxidant capacity of ascorbic acid (AA) with 100 g of sample as follows:

AEAC (mg AA/100 g) = (IC_50AA_/IC50sample) × 100000 (2)

#### 3.2.2. Reducing Power

Reducing power was measured according to the method of Wang et al. [6]. Briefly, 20 µL of the sample was mixed with 50 µL of phosphate buffer (0.2 M; pH 6.6) and 50 µL of potassium ferricyanide (1%). The mixture was incubated at 50 °C for 20 min, and 50 µL of trichloroacetic acid (30%) was added to the reaction followed by a centrifugation step (3000 min^−1^ for 15 min). Finally, 50 µL of the supernatant solution was mixed with 50 µL of deionized water and 10 µL of FeCl3 (0.1%), and then left to stand for 10 min. The absorbance was measured at 700 nm in a 96-well plate (200 μL) using xMark Microplate Spectrophotometer (BioRad, Hercules, CA, USA).

The results were then expressed in terms of quercetin equivalent reducing capacity (QERC), which was calculated based on the reducing capacity at 0.5 absorbance unit of quercetin (QE) with 100 g of sample as follows:

QERC (mg QE/100 g) = (RC_0.5AU_ QE/RC_0.5AU_ sample) × 100000 (3)

### 3.3. Anti-Inflammatory Activity Assays

#### 3.3.1. Inhibition of Cyclooxygenase

The in vitro anti-inflammatory activity of fucoidan was examined using cyclooxygenase (COX-1 and COX-2) inhibition assays. The inhibition of human recombinant cyclooxygenase COX-1 and COX-2 (Cayman Chemical, Ann Arbor, MI, USA) was assessed according to the manufacturer’s instructions. Indomethacin (1 μg mL^−1^) from Sigma (St Louis, MO, USA) was used as reference. Fucoidan was dissolved in water prior to analysis.

#### 3.3.2. Hyaluronidase Activity

Hyaluronidase activity was assayed as described previously using a modified version of the Morgan–Elson method [75]. Briefly, 100 μL of the hyaluronic acid (Sigma-Aldrich Co., St Louis, MO, USA) solution was mixed with 200 μL of buffer at a pH of 3.85, and 500 μL of water in test tubes. The reaction was started by the addition of 100 μL of hyaluronidase (from bovine, Type IV-S, Sigma-Aldrich Co., St Louis, MO, USA) with 200 μL of reference or sample and followed with incubation at 37 °C for 10 min. The enzymatic reaction was stopped by adding of 110 μL of alkaline borate solution and subsequent heating for 4.5 min in a boiling water bath. The test tubes were then placed on ice for 30 min, and 3.0 mL of *p*-dimethylaminobenzaldehyde solution was added. The tubes were incubated at 37 °C for 20 min. After centrifugation, the supernatant absorbance was measured with a PharmaSpec 1700 spectrophotometer (Shimadzu, Kyoto, Japan) at 584 nm. Test samples were replaced by the buffer solution for the control, while the enzyme solution was replaced by a buffer solution for the blank. The buffer was prepared by dissolving of 0.68 g sodium formate, 0.29 g NaCl, and 0.01 g bovine serum albumin (BSA) in 45 mL of water. The pH (2.0–5.0) was adjusted with formic acid.

The percentage of inhibition was calculated according to the following equation:

Inhibition (%) = [(A−B) − (C−D)]/(A−B) × 100(4)

where A is the absorbance of the control, B is the absorbance of the control blank, C is the absorbance of the sample, and D is the absorbance of the sample blank. The IC_50_ was calculated using the mean of three observations from each of the concentrations.

#### 3.3.3. Cell Lines and Cell Culture

The human mononuclear U937 cells were purchased from the Russian Collection of Cell Culture (Institute of Cytology of Russian Academy of Science, Saint-Petersburg, Russia), and maintained at 37 °C in a humidified 95% air and 5% CO_2_ atmosphere in RPMI1640 supplemented with 2 mM glutamine, 10% heat-inactivated FBS, 100 U mL^−1^ penicillin, and 100 μg mL^−1^ streptomycin. Fucoidan was dissolved in water as a stock solution at a 10 mg mL^−1^ concentration, and the stock solution was then diluted with the medium to the desired concentration prior to use. Cells derived from the freeze-down batch were thawed, grown and seeded (106 cells per well) onto 12-well tissue culture plates and cultured in medium for 24 h. The cells were then stimulated with 1 μg mL^−1^ *Escherichia coli* LPS (Sigma-Aldrich, St. Louis, MO, USA) at 37 °C for 1 h. After that, the cells were treated with SB203580 (Sigma-Aldrich, St. Louis, MO, USA) and various concentrations of fucoidan at 37 °C for 1 h.

#### 3.3.4. Western Blotting

Cells were washed in cold (4 °C) phosphate-buffered saline (PBS; 0.5 mol L^−1^ sodium phosphate, pH 7.5) and separated by centrifugation (Hermle Labortechnik, Germany) at 1500 rpm^−1^ for 5 min at 4 °C, harvested by gentle scraping, and used to prepare total protein or nuclear extracts. Cells were treated with lysis buffer (1 mol L^−1^ Tris-HCl pH 7.5, 1.5 mol L^−1^ NaCl, 10% Triton X-100, 0.2 mol L^−1^ Na_3_VO_4_, 1 mol L^−1^ NaF, 0.2 mol L^−1^ EDTA, phenylmethylsulphonyl fluoride (PMSF), Abcam’s protease inhibitor cocktail, and Abcam’s phosphatase inhibitor cocktail) for 20 min at 4 °C. The lysates were then clarified by centrifugation at 15000 rpm^−1^ for 15 min at 4 °C and the supernatant was collected.

The protein concentrations of the extracts were determined using spectrophotometry XMark (Bio-Rad, USA). For Western blot analysis, 40 μg of protein was desaturated by boiling with Laemmli buffer (5 min at 100 °C) and subjected to 4%–14% SDS-polyacrylamide gels, and transferred to nitrocellulose membranes (Bio-Rad, USA) by electroblotting. The membranes were blocked with 5% non-fat dry milk in PBS with Tween 20 buffer (PBS-T) (Tris-HCl (pH 7.5), 1.5 mol NaCl, and 0.1% Tween 20) for 1 h at room temperature. Membranes were then incubated overnight at 4 °C with the primary antibodies, probed with enzyme-linked secondary antibodies, and visualized using a chemiluminescent detection with LumiGLO^®^ reagent (Cell Signaling Technology, Danvers, MA, USA) according to the manufacturer’s instructions. After detection the membranes were scanned (Epson Perfection V330 Photo) and processed with Scion Image software (Alpha 4.0.3.2, National Institutes of Health, USA). The band intensities were used for calculations. Рhospho-р38 МАРК antibody rabbit, p38 МАРК XP rabbit mAb, β-actin rabbit mAb, and anti-rabbit IgG, HRP-linked antibody were from Cell Signaling Technology (USA). SB203580 (5 µM (1.88 µg mL^−1^ final concentration) was used as the positive control.

### 3.4. Anti-Hyperglycemic Activity Assay

The inhibition of human DPP-IV (DPP-IV, Sigma-Aldrich) was assessed by using a chromogenic substrate method with glycine-proline-p-nitroanilide (Gly-Pro-PNA) as a substrate.

To each well of a 96-well microtiter plate, the enzyme (20 μL, 0.1 IU mL^−1^), substrate (40 μL, 1 mM), and 40 μL of Tris buffer (0.1 M, pH 8.0) were added in turn and mixed. Volumes of 20 μL of positive control (4 μg mL^−1^), negative control or sample (0.2–200 μg mL^−1^) were added, and the plate was incubated at 37 °C for 30 min. The absorbance of each well was measured at 405 nm every 5 min with microplate Spectrophotometer xMarkTM (Bio-Rad Laboratories, Inc., Hercules, CA, USA). Each test was performed with five replicates, and the result was given as an average. The test assay was validated with sitagliptin—a well-known synthetic inhibitor of DPP-IV.

### 3.5. Anti-Coagulant Activity Assay

The activated partial thromboplastin time (APTT) was measured according to the method of L.O. Anderson et al. [76] with minor modification. Platelet-poor plasma samples (0.05 mL) were mixed with different amounts of fucoidan from *F. vesiculosus* in water (0.015 mL) solution and incubated for 60 s at 37 °C. Subsequently, 0.05 mL of prewarmed APTT reagent was added and the mixture was allowed to incubate for 3 min at 37 °C. Prewarmed 0.25 mol L^−1^ calcium chloride (0.05 mL) was then added, and the APTT was determined by semiautomatic blood coagulation analyzer in a coagulometer (APG2-02-P, LLC EMCO, Moscow, Russia). Water and solutions of heparin were used as negative and positive controls, respectively.

Prothrombin time (PT) was determined according to the method of Quick [77] with minor modification. The reaction mixture containing different concentrations of fucoidan was incubated with 0.05 mL of plasma for 3 min at 37 °C; then, prewarmed PT reagent (0.1 mL) was added, and the time for clot formation was determined by semiautomatic blood coagulation analyzer. Water and solutions of heparin were used as negative and positive controls, respectively.

Thrombin time (TT) was determined using the method of Denson and Wang [15,78] with minor modification. Plasma samples (0.05 mL) were mixed with fucoidan at different concentration levels in 0.9% NaCl (0.05 mL) solution. Subsequently, 0.05 mL of prewarmed TT reagent was added, and the time for clot formation was determined by a semiautomatic blood coagulation analyzer. Water and solutions of heparin were used as negative and positive controls, respectively.

### 3.6. Statistical Analysis

All experimental results were expressed as the mean of triplicate ± standard deviation (SD). Data were analyzed using Statistica version 6.0. Significant differences were considered as *p* < 0.05.

## 4. Conclusions

A biological evaluation of fucoidan from *F. vesiculosus* with a high Mw and sulfate content is presented for the first time in this work. Our data indicated that the beneficial effects of fucoidan rely on a variety of cellular and molecular mechanisms such as radical scavenging, down-regulation of COX-1/2, MAPK p38, inhibition of hyaluronidase, DPP-IV and extension of APTT and TT. As far as we know, the inhibition of hyaluronidase and DPP-IV by high molecular fucoidan was studied for the first time in this work. Our results and literature data suggest that molecular weight, sulfate content, fucose content, and polyphenols may contribute to these activities. High molecular weight fucoidan may have promising therapeutic applications in different pharmacological settings.

Interestingly, anti-oxidant, anti-inflammatory and anti-coagulant drugs have been used for the management of COVID19 complications. It has been suggested that a proper dose of anti-oxidants may ameliorate the cardiac injuries of critically ill patients with COVID19 [79]. Chinese scientists have come to the conclusion that anti-inflammatory treatment, started at the right time, is crucial in the therapy of patients with COVID19 [80]. Prophylactic doses of low-Mw heparins are recommended for all patients who require hospitalization for the management of COVID19 coagulopathy [81]. In summary, fucoidan could be considered as a prospective candidate for amelioration the treatment of patients with COVID19; however, additional research in this field is required.

## Figures and Tables

**Table 1:** Comparison of 1, 1-Diphenyl-2-picryl hydrazil (DPPH) radical scavenging activities of the fucoidan extracted from *F. vesiculosus*, and reference compounds, Mean ± SD, (n = 3). AEAC: ascorbic acid equivalent anti-oxidant capacity.

Sample	IC_50_ (mg mL^−1^)	AEAC (mgAA/100 g)
Fucoidan	0.035 ± 0.002	914 ± 28
Quercetin	0.026 ± 0.001	1231 ± 56
Butylated hydroxyanisole (BHA)	0.00059 ± 0.00005	54237 ± 22
Butylated hydroxytoluene (BHT)	0.00092 ± 0.00003	34783 ± 14
Ascorbic acid (AA)	0.00032 ± 0.00002	

**Table 2:** Effect of fucoidan on the phosphorylation of mitogen-activated protein kinase (MAPK) p38 in the human mononuclear U937 cells stimulated with lipopolysaccharides (LPS). Mean ± SEM, (n = 6).

Sample, Concentration	Percentage of MAPK p38 (%)
Intact cells (no stimulation with LPS)	23.0 ± 1.2
Control cells stimulated with LPS (1 μg mL^−1^)	100
SB203580 (1.88 μg mL^−1^) + LPS	41.0 ± 1.8
Fucoidan (0.25 μg mL^−1^) + LPS	17.0 ± 0.6
Fucoidan (0.125 μg mL^−1^) + LPS	20.0 ± 0.5
Fucoidan (0.05 μg mL^−1^) + LPS	31.0 ± 0.9
Fucoidan (0.025 μg mL^−1^) + LPS	53.0 ± 0.8
Fucoidan (0.0125 μg mL^−1^) + LPS	56.0 ± 0.9
Fucoidan (0.00625 μg mL^−1^) + LPS	77.0 ± 0.9

## References

[1] Mayer A.M.S., Guerrero A.J., Rodríguez A.D., Taglialatela-Scafati O., Nakamura F., Fusetani N. (2020). Marine pharmacology in 2014–2015: Marine compounds with antibacterial, antidiabetic, antifungal, anti-inflammatory, antiprotozoal, antituberculosis, antiviral, and anthelmintic activities; affecting the immune and nervous systems, and other miscellaneous mechanisms of action. Mar. Drugs.

[2] Usov A.I., Bilan M.I. (2009). Fucoidans—Sulfated polysaccharides of brown algae. Russ. Chem. Revs..

[3] Ushakova N.A., Morozevich G.E., Ustyuzhanina N.E., Bilan M.I., Usov A.I., Nifantiev N.E., Preobrazhenskaya M.E. (2008). Anticoagulant activity of fucoidans from brown algae. Biomed. Khim..

[4] Ale M.T., Mikkelsen J.D., Meyer A.S. (2011). Important determinants for fucoidan bioactivity: A critical review of structure-function relations and extraction methods for fucose-containing sulfated polysaccharides from brown seaweeds. Mar. Drugs.

[5] Ale M.T., Meyer A.S. (2013). Fucoidans from brown seaweeds: An update on structures, extraction techniques and use of enzymes as tools for structural elucidation. RSC Adv..

[6] Wang J., Zhang Q., Zhang Z., Li Z. (2008). Antioxidant activity of sulfated polysaccharide fractions extracted from *Laminaria japonica*. Int. J. Biol. Macromol..

[7] Wang Y., Xing M., Cao Q., Ji A., Liang H., Song S. (2019). Biological activities of fucoidan and the factors mediating its therapeutic effects: A review of recent studies. Mar. Drugs.

[8] Koh H.S.A., Lu J., Zhou W. (2019). Structure characterization and antioxidant activity of fucoidan isolated from *Undaria pinnatifida* grown in New Zealand. Carbohydr. Polym..

[9] Sanjeewa K.A., Jayawardena T.U., Kim H.-S., Kim S.Y., Fernando I.P.S., Wang L., Abetunga D.T.U., Kim W.-S., Lee D.-S., Jeon Y.-J. (2019). Fucoidan isolated from *Padina commersonii* inhibit LPS-induced inflammation in macrophages blocking TLR/NF-κB signal pathway. Carbohydr. Ppolym..

[10] Fernando I.S., Sanjeewa K.A., Samarakoon K.W., Lee W.W., Kim H.-S., Ranasinghe P., Gunasekara U.K.D.S.S., Jeon Y.J. (2018). Antioxidant and anti-inflammatory functionality of ten Sri Lankan seaweed extracts obtained by carbohydrase assisted extraction. Food Sci. Biotechnol..

[11] Yang X.D., Liu C.G., Tian Y.J., Gao D.H., Li W.S., Ma H.L. (2017). Inhibitory effect of fucoidan on hypoglycemia in diabetes mellitus anim. Int. J. Clin. Exp. Med..

[12] Fernando I.S., Ryu B., Ahn G., Yeo I.K., Jeon Y.J. (2020). Therapeutic potential of algal natural products against metabolic syndrome: A review of recent developments. Trends Food Sci. Technol..

[13] Irhimeh M.R., Fitton J.H., Lowenthal R.M. (2009). Pilot clinical study to evaluate the anticoagulant activity of fucoidan. Blood Coagul. Fibrinolysis.

[14] Wang J., Zhang Q., Zhang Z., Song H., Li P. (2010). Potential antioxidant and anticoagulant capacity of low molecular weight fucoidan fractions extracted from *Laminaria japonica*. Int. J. Biol. Macromol..

[15] Wang J., Zhang Q.B., Zhang Z.S., Hou Z.S., Zhang H. (2011). In vitro anticoagulant activity of fucoidan derivatives from brown seaweed *Laminaria japonica*. Chin. J. Oceanol. Limnol..

[16] Zhao X., Guo F., Hu J., Zhang L., Xue C., Zhang Z., Li B. (2016). Antithrombotic activity of oral administered low molecular weight fucoidan from *Laminaria japonica*. Thromb. Res..

[17] Krylova N.V., Ermakova S.P., Lavrov V.F., Leneva I.A., Kompanets G.G., Iunikhina O.V., Nosik M.N., Ebralidze L.K., Falynskova I.N., Silchenko A.S. (2020). The comparative analysis of antiviral activity of native and modified fucoidans from brown algae *Fucus evanescens* in vitro and in vivo. Mar. Drugs.

[18] Pozharitskaya O.N., Shikov A.N., Faustova N.M., Obluchinskaya E.D., Kosman V.M., Vuorela H., Makarov V.G. (2018). Pharmacokinetic and tissue distribution of fucoidan from *Fucus vesiculosus* after oral administration to rats. Mar. Drugs.

[19] Fitton J.H., Stringer D.N., Park A.Y., Karpiniec S.S. (2019). Therapies from fucoidan: New developments. Mar. Drugs.

[20] Oka S., Okabe M., Tsubura S., Mikami M., Imai A. (2020). Properties of fucoidans beneficial to oral healthcare. Odontology.

[21] Fitton J.H., Dell’Acqua G., Gardiner V.-A., Karpiniec S.S., Stringer D.N., Davis E. (2015). Topical benefits of two fucoidan-rich extracts from marine macroalgae. Cosmetics.

[22] Pozharitskaya O.N., Shikov A.N., Obluchinskaya E.D., Vuorela H. (2019). The pharmacokinetics of fucoidan after topical application to rats. Mar. Drugs.

[23] Chakraborty K., Joseph D., Joy M., Raola V.K. (2016). Characterization of substituted aryl meroterpenoids from red seaweed *Hypnea musciformis* as potential antioxidants. Food Chem..

[24] Zhang Y.J., Gan R.Y., Li S., Zhou Y., Li A.N., Xu D.P., Li H.B. (2015). Antioxidant phytochemicals for the prevention and treatment of chronic diseases. Molecules.

[25] Holgate S.T., Peters-Golden M., Panettieri R.A., Henderson W.R. (2003). Roles of cysteinyl leukotrienes in airway inflammation, smooth muscle function, and remodeling. J. Allergy Clin. Immunol..

[26] Kumar K.A. (2011). High-through screening assays for cyclooxygenase-2 and 5-lipoxygenase, the targets for inflammatory disorders. Ind. J. Biochem. Biophys..

[27] Saklatvala J. (2004). The p38 MAP kinase pathway as a therapeutic target in inflammatory disease. Curr. Opin. Pharmacol..

[28] Yang S.H., Sharrocks A.D., Whitmarsh A.J. (2003). Transcriptional regulation by the MAP kinase signaling cascades. Gene.

[29] Katsube T., Yamasaki Y., Iwamoto M., Oka S. (2003). Hyaluronidase-inhibiting polysaccharide isolated and purified from hot water extract of sporophyll of *Undaria pinnatifida*. Food Sci. Technol. Res..

[30] Elzoghby A.O., Freag M.S., Elkhodairy K.A. (2018). Biopolymeric nanoparticles for targeted drug delivery to brain tumors. Nanotechnology-Based Targeted Drug Delivery Systems for Brain Tumors.

[31] Havale S.H., Pal M. (2009). Medicinal chemistry approaches to the inhibition of dipeptidyl peptidase-4 for the treatment of type 2 diabetes. Bioorg. Med. Chem..

[32] Shan X.D., Liu X., Hao J.J., Cai C., Fan F., Dun Y.L., Zhao X.L., Liu X.X., Li C.X., Yu G.L. (2016). In vitro and in vivo hypoglycemic effects of brown algal fucoidans. Int. J. Biol. Macromol..

[33] Dürig J., Bruhn T., Zurborn K.-H., Gutensohn K., Bruhn H.D., Béress L. (1997). Anticoagulant fucoidan fractions from fucus vesiculosus induce platelet activation in vitro. Thromb. Res..

[34] Zayed A., Muffler K., Hahn T., Rupp S., Finkelmeier D., Burger-Kentischer A., Ulber R. (2016). Physicochemical and biological characterization of fucoidan from *Fucus vesiculosus* purified by dye affinity chromatography. Mar. Drugs.

[35] Lahrsen E., Schoenfeld A.K., Alban S. (2018). Size-dependent pharmacological activities of differently degraded fucoidan fractions from *Fucus vesiculosus*. Carbohydr. Polym..

[36] Jönsson M., Allahgholi L., Sardari R.R., Hreggviðsson G.O., Nordberg Karlsson E. (2020). Extraction and modification of macroalgal polysaccharides for current and next-generation applications. Molecules.

[37] Luthuli S., Wu S., Cheng Y., Zheng X., Wu M., Tong H. (2019). Therapeutic effects of fucoidan: A review on recent studies. Mar. Drugs.

[38] Fletcher H.R., Biller P., Ross A.B., Adams J.M.M. (2017). The seasonal variation of fucoidan within three species of brown macroalgae. Algal Res..

[39] Seyoum A., Asres K., El-Fiky F.K. (2006). Structure–radical scavenging activity relationships of flavonoids. Phytochemistry.

[40] Lim S.J., Aida W.M.W., Maskat M.T., Mamot S., Ropien J., Mohd D.M. (2014). Isolation and antioxidant capacity of fucoidan from selected Malaysian seaweeds. Food Hydrocoll..

[41] Yuan Y., Macquarrie D. (2015). Microwave assisted extraction of sulfated polysaccharides (fucoidan) from ascophyllum nodosum and its antioxidant activity. Carbohydr. Polym..

[42] Rodriguez-Jasso R., Mussatto S., Pastrana L., Aguilar C., Teixeira J. (2014). Chemical composition and antioxidant activity of sulphated polysaccharides extracted from *Fucus vesiculosus* using different hydrothermal processes. Chem. Pap..

[43] Le K., Chiu F., Ng K. (2007). Identification and quantification of antioxidants in *Fructus lycii*. Food Chem..

[44] He J., Xu Y., Chen H., Sun P. (2016). Extraction, structural characterization, and potential antioxidant activity of the polysaccharides from four seaweeds. Int. J. Mol. Sci..

[45] Phull A.R., Kim S.J. (2017). Fucoidan as bio-functional molecule: Insights into the antiinflammatory potential and associated molecular mechanisms. J. Funct. Foods.

[46] Zayed A., Ulber R. (2020). Fucoidans: Downstream processes and recent applications. Mar. Drugs.

[47] Yang Y., Liu D., Wu J., Chen Y., Wang S. (2011). In vitro antioxidant activities of sulfated polysaccharide fractions extracted from *Corallina officinalis*. Int. J. Biol. Macromol..

[48] Zayed A., Hahn T., Finkelmeier D., Burger-Kentischer A., Rupp S., Krämer R., Ulber R. (2019). Phenomenological investigation of the cytotoxic activity of fucoidan isolated from *Fucus vesiculosus*. Process Biochem..

[49] Álvarez-Viñas M., Flórez-Fernández N., González-Muñoz M.J., Domínguez H. (2019). Influence of molecular weight on the properties of *Sargassum muticum* fucoidan. Algal Res..

[50] Mak W., Hamid N., Liu T., Lu J., White W.L. (2013). Fucoidan from New Zealand *Undaria pinnatifida*: Monthly variations and determination of antioxidant activities. Carbohydr. Polym..

[51] Somasundaram S.N., Shanmugam S., Subramanian B., Jaganathan R. (2016). Cytotoxic effect of fucoidan extracted from *Sargassum cinereum* on colon cancer cell line HCT-15. Int. J. Biol. Macromol..

[52] Wang T., Jónsdóttir R., Ólafsdóttir G. (2009). Total phenolic compounds, radical scavenging and metal chelation of extracts from Icelandic seaweeds. Food Chem..

[53] Parys S., Kehraus S., Krick A., Glombitza K.W., Carmeli S., Klimo K., Gerhauser C., Konig G.M. (2010). In vitro chemopreventive potential of fucophlorethols from the brown alga *Fucus vesiculosus* L. by anti-oxidant activity and inhibition of selected cytochrome P450 enzymes. Phytochemistry..

[54] Jouzeau J.Y., Terlain B., Abid A., Nédélec E., Netter P. (1997). Cyclo-oxygenase isoenzymes. How recent findings affect thinking about nonsteroidal anti-inflammatory drugs. Drugs.

[55] Smith W.L., DeWitt D.L., Garavito R.M. (2000). Cyclooxygenases: Structural, cellular, and molecular biology. Annu. Rev. Biochem..

[56] Park H.Y., Han M.H., Park C., Jin C.Y., Kim G.Y., Choi I.W., Kim N.D., Nam T.J., Kwon T.K., Choi Y.H. (2011). Anti-inflammatory effects of fucoidan through inhibition of NF-κB, MAPK and Akt activation in lipopolysaccharide-induced BV2 microglia cells. Food Chem. Toxicol..

[57] Dewi L. (2016). In silico analysis of the potential of the active compounds fucoidan and alginate derived from *Sargassum* sp. as inhibitors of COX-1 and COX-2. Med Arch..

[58] Lee S.H., Ko C.I., Ahn G., You S., Kim J.S., Heu M.S., Kim J., Jee Y., Jeon Y.J. (2012). Molecular characteristics and anti-inflammatory activity of the fucoidan extracted from *Ecklonia cava*. Carbohydr. Polym..

[59] Phull A.R., Majid M., Haq I.U., Khan M.R., Kim S.J. (2017). In vitro and in vivo evaluation of anti-arthritic, antioxidant efficacy of fucoidan from *Undaria pinnatifida* (Harvey) Suringar. Int. J. Biol. Macromol..

[60] Lim J.D., Lee S.R., Kim T., Jang S.-A., Kang S.C., Koo H.J., Sohn E., Bak J.P., Namkoong S., Kim H.K. (2015). Fucoidan from *Fucus vesiculosus* protects against alcohol-induced liver damage by modulating inflammatory mediators in mice and HepG2 cells. Mar. Drugs.

[61] Jayawardena T.U., Fernando I.P.S., Lee W.W., Sanjeewa K.K.A., Kim H.S., Lee D.S., Jeon Y.J. (2019). Isolation and purification of fucoidan fraction in *Turbinaria ornata* from the Maldives; Inflammation inhibitory potential under LPS stimulated conditions in in-vitro and in-vivo models. Int. J. Biol. Macromol..

[62] Ministry of Public Health and Social Development of the Russian Federation (2018). Online State Register of Medicinal Preparations. http://grls.rosminzdrav.ru/Grls_View_v2.aspx?routingGuid=e8ad7e7f-98c1-4389-a6d6-1fc8c4cd969a&t=.

[63] Rosoiu N., Nita R., Olariu L., Drumea V., Ene D.M. (2008). Original bioactive complexes rich in glycosaminoglycans obtained from small fish. Roum. Soc. Biol. Sci..

[64] Kim K.J., Lee O.H., Lee B.Y. (2010). Fucoidan, a sulfated polysaccharide, inhibits adipogenesis through the mitogen-activated protein kinase pathway in 3T3-L1 preadipocytes. Life Sci..

[65] Sanjeewa K.K., Fernando I.P., Kim E.A., Ahn G., Jee Y., Jeon Y.J. (2017). Anti-inflammatory activity of a sulfated polysaccharide isolated from an enzymatic digest of brown seaweed *Sargassum horneri* in RAW 264.7 cells. Nutr. Res. Pract..

[66] Unnikrishnan P.S., Suthindhiran K., Jayasri M.A. (2014). Inhibitory potential of *Turbinaria ornata* against key metabolic enzymes linked to diabetes. BioMed Res. Int..

[67] Unnikrishnan P.S., Suthindhiran K., Jayasri M.A. (2015). Antidiabetic potential of marine algae by inhibiting key metabolic enzymes. Front. Life Sci..

[68] Wheeler A.P., Gailani D. (2016). The intrinsic pathway of coagulation as a target for antithrombotic therapy. Hematol. Oncol. Clin. North Am..

[69] Chen X., Yang S., Wang J., Song L., Xing R., Liu S., Li P. (2015). Sulfated polysaccharides isolated from cloned *Grateloupia filicina* and their anticoagulant activity. BioMed Res. Int..

[70] Athukorala Y., Jung W.K., Park P.J., Lee Y.J., Kim S.K., Vasanthan T., Jeon Y.J. (2008). Evaluation of biomolecular interactions of sulfated polysaccharide isolated from *Grateloupia filicina* on blood coagulation factors. J. Microbiol. Biotechnol..

[71] Zhang Z., Till S., Knappe S., Quinn C., Catarello J., Ray G.J., Scheiflinger F., Szabo C.M., Dockal M. (2015). Screening of complex fucoidans from four brown algae species as procoagulant agents. Carbohydr. Polym..

[72] Obluchinsksya E.D., Makarova M.N., Pozharitskaya O.N., Shikov A.N. (2015). Effects of ultrasound treatment on the chemical composition and anticoagulant properties of dry *Fucus* extract. Pharm. Chem. J..

[73] Thanh T.T.T., Tran V.T.T., Yuguchi Y., Bui L.M., Nguyen T.T. (2013). Structure of fucoidan from brown seaweed *Turbinaria ornata* as studied by electrospray ionization mass spectrometry (ESIMS) and small angle X-ray scattering (SAXS) techniques. Mar. Drugs.

[74] Imbs T.I., Skriptsova A.V., Zvyagintseva T.N. (2015). Antioxidant activity of fucose-containing sulfated polysaccharides obtained from *Fucus evanescens* by different extraction methods. J. Appl. Phycol..

[75] Akbarov U.S., Pozharitskaya O.N., Laakso I., Seppänen-Laakso T., Urakova I.N., Vuorela H., Makarov V.G., Shikov A.N. (2020). Metabolite profiling and mechanisms of bioactivity of snake autolysate—A traditional Uzbek medicine. J. Ethnopharmacol..

[76] Anderson L.O., Barrowcliffe T.W., Holmer E., Johnson E.A., Sims G.E.C. (1976). Anticoagulant properties of heparin fractionated by affinity chromatography on matrix-bound antithrombin III and by gel filtration. Thromb. Res..

[77] Quick A.J. (1940). The clinical application of the hippuric acid and the prothrombin tests. Am. J. Clin. Pathol..

[78] Denson K.W., Bonnar J. (1973). The measurement of heparin: A method based on the potentiation of anti-factor Xa. Thromb. Haemost..

[79] Wang J.Z., Zhang R.Y., Bai J. (2020). An anti-oxidative therapy for ameliorating cardiac injuries of critically ill COVID-19-infected patients. Int. J. Cardiol..

[80] Zhang W., Zhao Y., Zhang F., Wang Q., Li T., Liu Z., Wang J., Qin Y., Zhang X., Yan X. (2020). The use of anti-inflammatory drugs in the treatment of people with severe coronavirus disease 2019 (COVID-19): The experience of clinical immunologists from China. Clin. Immunol..

[81] Thachil J., Tang N., Gando S., Falanga A., Cattaneo M., Levi M., Clarck C., Iba T. (2020). ISTH interim guidance on recognition and management of coagulopathy in COVID-19. J. Thromb. Haemost..

